# Neddylation regulation of mitochondrial structure and functions

**DOI:** 10.1186/s13578-021-00569-6

**Published:** 2021-03-17

**Authors:** Qiyin Zhou, Yawen Zheng, Yi Sun

**Affiliations:** 1 Cancer Institute, The Second Affiliated Hospital, and Institute of Translational Medicine, Zhejiang University School of Medicine, Hangzhou, 310029 Zhejiang China; 2 Department of Medical Oncology, Sir Run Run Shaw Hospital, Zhejiang University School of Medicine, Hangzhou, 310016 Zhejiang China

**Keywords:** Cullin-RING ligases, Energy metabolism, Mitochondria, MLN4924, Neddylation

## Abstract

Mitochondria are the powerhouse of a cell. The structure and function of mitochondria are precisely regulated by multiple signaling pathways. Neddylation, a post-translational modification, plays a crucial role in various cellular processes including cellular metabolism via modulating the activity, function and subcellular localization of its substrates. Recently, accumulated data demonstrated that neddylation is involved in regulation of morphology, trafficking and function of mitochondria. Mechanistic elucidation of how mitochondria is modulated by neddylation would further our understanding of mitochondrial regulation to a new level. In this review, we first briefly introduce mitochondria, then neddylation cascade, and known protein substrates subjected to neddylation modification. Next, we summarize current available data of how neddylation enzymes, its substrates (including cullins/Cullin-RING E3 ligases and non-cullins) and its inhibitor MLN4924 regulate the structure and function of mitochondria. Finally, we propose the future perspectives on this emerging and exciting field of mitochondrial research.

## Introduction

Mitochondria, the highly dynamic and semi-autonomous organelles wrapped with a double membrane, are deeply integrated into cellular signaling pathways and play the essential role in regulation of a variety of metabolisms such as energy production, calcium homeostasis, and reactive oxidative species (ROS) balance [1, 2]. Moreover, mitochondria regulate various essential cellular physiological processes such as differentiation, cell pluripotency and cell death [3]. Consequently, dysfunctional mitochondria have been observed in many pathological conditions including cancer, cardiovascular disorders, and metabolic diseases [4–7]. Thus, to maintain structural and functional integrity, and the well-being of a cell, mitochondria are subjected to fine regulations at the multiple levels under various physiological and pathological conditions [8–15].

**Fig. 1:**
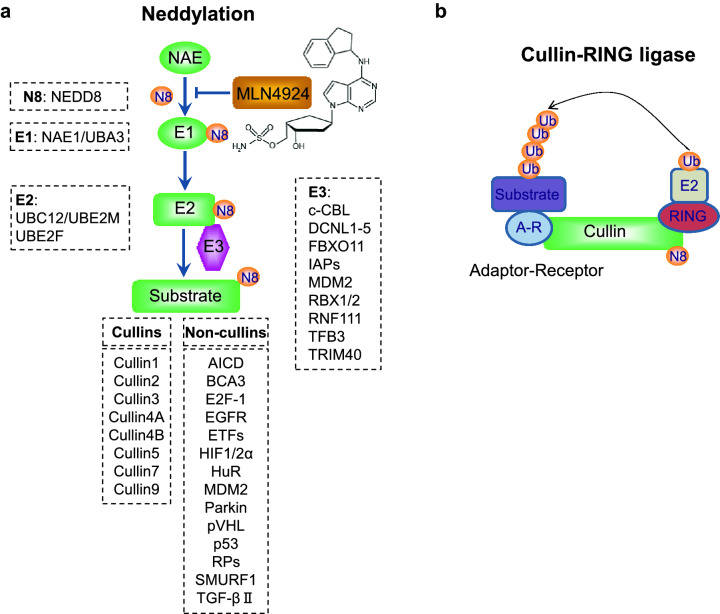
The process of neddylation modification. **a** Neddylation is a process that tags the ubiquitin-like small molecule NEDD8 onto its substrate through an enzymatic cascade involving NEDD8-activating enzyme E1, NEDD8-conjugating enzyme E2 and substrate-specific NEDD8 E3 ligases. MLN4924 is a NAE inhibitor that blocks the entire neddylation pathway. **b** Cullin-RING ligase, consisting of a scaffold cullin, a RING protein that binds to NEDD8-loaded E2, an adaptor, and a substrate receptor, promotes ubiquitylation and degradation of their substrates. N8, NEDD8

Protein neddylation is an important posttranslational modification in eukaryotes. To date, neddylation modification has been well-established to tag the extremely well conserved neuronal precursor cell-expressed developmentally down-regulated protein8 (NEDD8) onto the substrates to modulate their function, subcellular localization, and activity [16]. Recently, a study also showed that NEDD8 conjugates to SRSF3 on lysine 11 for poly-neddylation and subsequent proteasome-mediated degradation [17]. Similar to ubiquitylation, NEDD8 is first activated in an ATP-dependent manner by one heterodimeric E1 NEDD8-activating enzyme (NAE, NAE1/APPBP1 and NAEβ/UBA3), then transferred to one of the two NEDD8 conjugating enzymes (UBE2M/UBC12 and UBE2F) through a trans-thiolation reaction, and finally conjugated to target substrates catalyzed by one of dozen E3 neddylation ligases [18] (Fig. 1a).

To date, the best-characterized neddylation substrates are the cullin (CUL) family proteins, including CUL1, CUL2, CUL3, CUL4A, CUL4B, CUL5, CUL7, and CUL9. Cullin is the scaffold component, which complexes with other components, including an adaptor, a substrate receptor and a RING component, to form Cullin-RING ligases (CRLs) [16] (Fig. 1b). The conjugation of NEDD8 to a cullin activates CRL E3 ligase, the largest family of E3 ubiquitin ligases, which is responsible for the ubiquitylation of about 20% cellular proteins for targeted degradation through ubiquitin proteasome system (UPS) [19]. Therefore, neddylation precisely controls many biological processes through CRLs-mediated ubiquitylation and degradation upon neddylation activation [20, 21]. The overactivation of neddylation modification and abnormal expression of CRL components have been found in many human diseases, particularly human cancers [22]. MLN4924, also known as pevonedistat, binds to the active site of NAE catalytic subunit and forms a covalent NEDD8-MLN4924 adduct, which resembles adenylated NEDD8, the first intermediate in the NAE reaction cycle, but cannot be further utilized in subsequent intraenzyme reactions [23]. Therefore, MLN4924 directly inhibits the entire neddylation modification and indirectly inhibits the CRLs [19] (Fig. 1a). As a result, MLN4924 treatment triggers various cellular responses such as cell cycle arrest, apoptosis, senescence, autophagy as well as metabolic reprogramming [19, 24, 25]. Given that preclinical studies both in vitro cell culture settings and in vivo xenograft models showed potent antitumor activity and well-tolerated toxicity, MLN4924 has been advanced into several phase II clinical trials for anticancer therapy as a single agent or in combination with chemotherapeutic drugs [22].

The neddylation has been implicated in various metabolic processes, including adipogenesis, lipid droplet formation, and redox homeostasis [26–31]. Interestingly, emerging data revealed that mitochondrial functions are subjected to the regulation by neddylation and CRLs. More importantly, different approaches including mitochondria purification and confocal microscopy image analysis have shown that mitochondria contain various components of neddylation and CRLs, as well as non-cullin substrates, providing molecular basis for such a regulation [28, 32, 33].

## Mitochondrial regulation by neddylation enzymes

### NEDD8 activating enzyme (NAE)

UBA is the catalytic subunit of NAE [34], and a direct drug target of MLN4924 [19]. Our recent study showed that like MLN4924 (see below), UBA3 knockdown also markedly induces mitochondrial fission-to-fusion conversion to form filamental mitochondria [35]. In addition, one study reported that UBA3 is involved in mitochondrial respiration, since UBA3-deficient neonatal primary hepatocytes manifest reduced basal and maximal respiration, as compared to UBA3-sufficient ones [28].

### NEDD8 conjugating enzymes

While there is no direct report to show that mitochondrial structure or function is subjected to regulation by neddylation E2s (Ube2M/UBC12 and Ube2F), our recent study found a negative cross-talk between UBE2M and UBE2F. Specifically, UBE2M complexes with Parkin-DJ1, an E3 localized in mitochondria under stressed conditions to promote ubiquitylation and degradation of UBE2F [21]. However, the biochemical and biological significances as to how this degradation impacts mitochondrial function remain elusive.

### NEDD8 ligases

Two well-defined neddylation co-E3s, RBX1 and RBX2 (also known as SAG for Sensitive to Apoptosis Gene) were found to regulate mitochondrial functions [36, 37]. One study showed that upon mitochondrial damage, RBX1 promotes ubiquitylation and degradation of Suppression of Sestrin 2 (SESN2) to trigger the generation of mitochondrial ROS, leading to cell death in neuroblastoma cells [38]. Another recent study showed that in mice hepatocytes with Drp1KO, Rbx1 is recruited to mitochondria in a p62-dependent manner to mediate mitochondrial ubiquitylation and subsequent mitophagy [32].

RBX2/SAG was originally cloned in our laboratory as a redox-inducible antioxidant protein [37], which scavenges oxidant at the expense of forming inter or intra molecular disulfide bond [39]. In mouse embryonic stem cells, Rbx2/Sag disruption increases the steady-state levels of ROS after exposure to ionizing radiation, leading to radiosensitization via enhanced apoptosis [40].

c-CBL is another neddylation E3 [41]. It was reported that the activity of several enzymes involved in mitochondrial fat oxidation and the phosphorylation of acetyl CoA carboxylases are significantly increased in the muscle tissues of c-Cbl deficient mice when fed with the high-fat diet [42]. However, the detailed underlying mechanism of c-CBL action is unknown, nor whether this is through its ubiquitylation or neddylation activity.

Collectively, very limited reports suggest that neddylation enzymes modulate mitochondrial structure and functions. Much more extensive studies with mechanistic elucidation are needed to firmly establish the notion that mitochondria are subjected to neddylation regulation via the enzymatic cascade.

## Mitochondrial regulation by neddylation substrates and CRLs

### Cullins

**Fig. 2:**
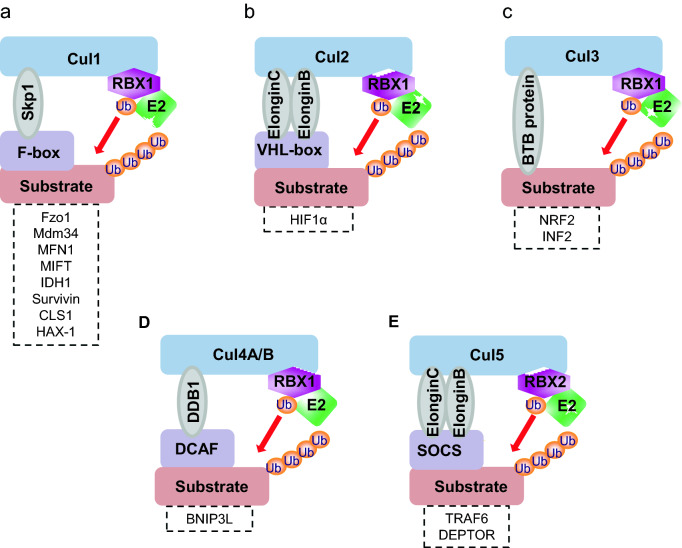
The modularity of Cullin-RING E3 ligases in mitochondria. Shown are the substrates of CRL1 (**a**), CRL2 (**b**), CRL3 (**c**), CRL4 (**d**), CRL5 (**e**) that are involved in the regulation of mitochondrial morphology and function

Cullins with eight family members are physiological substrates of neddylation [43]. Cullins are scaffold component of the cullin-RING ligases (CRLs), which are the largest family of E3 ubiquitin ligases, consisting of four subunits: a cullin with 8 family members, adaptor proteins with many members, substrate recognition receptors with many members and the RING component with two family members (RBX1/RBX2) [44] (Fig. 1b). Interestingly, RBX1/RBX2 serves as dual E3 for both ubiquitylation and neddylation. Cullin neddylation is required for activation of CRLs [43], and accumulated data have shown that CRLs are actively involved in regulation of morphology, trafficking, functions, and the degradation of mitochondria (Table 1 and Fig. 2).

**Table 1:** CRLs in regulation of mitochondrial functions

Category	Substrate receptors	Substrates	Regulatory functions/biological consequences	References
CRL1	Mdm30 (Yeast)	Fzo1	Inhibits Mitochondrial fusion	[46, 47]
		Mdm34	Inhibits Mitochondrial fusion	[48]
	Mfb1 (Yeast)	NR	Inhibits Mitochondrial fusion	[49]
	β-TrCP1	MFN1	Inhibits Mitochondrial fusion	[35]
	FBXW7	MIFT	Inhibits mitochondrial gene transcription and oxidative metabolism	[50]
	SKP2	IDH1	Inhibits tricarboxylic acid (TCA) cycle	[51]
	FBXL4	NR	Inhibits mitochondrial morphology, mtDNA integrity, and OXPHOS	[52–54, 56]
	FBXL7	Survivin	Inhibits mitochondrial morphology and membrane potential	[55]
	FBXO7	NR	Inhibits mitophagy	[59]
			Decreases mitochondrial membrane potential, ATP production, and oxygen consumption, increases cytosolic ROS production	[58]
	FBXO11	NR	Inhibits mitochondrial swelling	[57]
	FBXO15	CLS1	Inhibits mitochondrial function	[60]
	FBXO25	HAX-1	Induces apoptosis	[61]
CRL2	pVHL	HIF1α	Increases the rate of oxygen consumption	[64]
CRL3	Keap1	Nrf2	Promotes cellular oxidative stress and controls mitochondrial retrograde trafficking	[66, 67]
	SPOP	INF2	Inhibits mitochondrial fission	[68]
CRL4A	CRBN	NR	Acts specifically as a Lon-type protease in mitochondria	[70]
		BNIP3L	Inhibits mitophagy	[71]
	DCAF6	NR	Maintain sarcomere structure and mitochondrial/ contractile function in cardiomyocytes	[72]
CRL4B	AhR	NR	Promotes mitochondrial biogenesis against oxidative damage	[74]
			Suppresses mitochondrial dysfunction and apoptosis induced by cigarette smoke	[75]
CRL5	NR	TRAF6	Binds with SARM1 and recruited to PINK1 complexes on depolarized mitochondria and facilitates Parkin-induced mitophagy	[79]
	NR	DEPTOR	Suppresses mitochondrial respiration, mtDNA copy number, and citrate synthase activity	[82]

*NR* not reported

### CRL1

CRL1, also known as SCF (SKP1-Cullin 1-F box protein), is the best studied founding member of CRLs. The F-box protein, the CRL substrate recognition subunit, consists of 69 members in mammalian cells, which are classified into FBXW, FBXL, and FBXO subfamilies [44, 45]. In yeast, F-box protein Mdm30 was shown to target Fzo1 (an ortholog of mammalian mitofusion1/2) or Mdm34, two mitochondrial proteins for their turnover, leading to inhibition of mitochondrial fusion [46–48]. The mitochondrial dynamics is also regulated by Mfb1, another F-box protein, although its specific mitochondrial substrate(s) are yet to be identified [49].

In mammalian cells, our recent study showed that SCF^β−TrCP1^ E3 ligase, located in mitochondria, targeted MFN1 for ubiquitylation and degradation, and β-TrCP1 knockdown induced mitochondrial fusion due to MFN1 accumulation [35]. FBXW7 is well characterized F-box protein and its knockdown elevated the level of MITF, a lineage-specific master regulator of melanocytes to enhance mitochondrial transcriptional program and oxidative phosphorylation [50]. Furthermore, a recent study showed that SKP2, another well-characterized F-box protein, regulated the stability of IDH1/2, the key TCA cycle enzymes, and SKP2 knockdown promoted the TCA cycle by reducing glycolysis [51]. Other studies revealed that FBXL7 and FBXL4 modulated mitochondrial morphology and function through survivin and other uncharacterized substrates [52–56]. FBXO7 and FBXO11 (also characterized as a NEDD8 E3) were shown to regulate mitochondrial morphology and function, as well as mitochondrial elimination by modulating PARP activation, apoptosis, and mitophagy, respectively [57–59]. Finally, FBXO15 was found to modulate mitochondrial function through regulating the stability of CLS1 (cardiolipin synthase), an enzyme for generation of cardiolipin, a mitochondrial-specific lipid [60], while FBXO25 targeted mitochondrial HAX-1 for degradation to induce cellular apoptosis [61]. Thus, CRL1 regulates the structure and function of mitochondria at various aspects via modulating the stability of a variety of mitochondrial substrates.

### CRL2

CRL2 is a complex of cullin-2, RBX1, adaptor protein Elongin B/C and substrate recognition receptor VHL-box [62]. HIF1α is the best-known substrate of CRL2 [63]. It was recently reported that pVHL up-regulated (a) CHCHD4, a key component of the disulphide relay system in mitochondrial protein import within the inter-membrane space, and (b) respiratory chain subunits of complexes I and IV, leading to increased rate of oxygen consumption and alterations in glucose and glutamine metabolisms in renal cell carcinoma [64].

### CRL3

CRL3 is composed of cullin-3, RBX1, and BTB-containing substrate receptors, such as Kelch-like ECH-associated protein Keap1 and Speckle Type POZ protein SPOP [44, 65]. Nrf2 is the best-characterized substrate of CRL3-Keap1. In non-stress cells, Nrf2 is degraded by Keap1-mediated UPS. The oxidative stress and electrophiles, however, oxidize the cysteine residue in Keap1 to change its conformation, leading to blockage of NRF2 ubiquitylation and degradation. As result, NRF2 translocates to nucleus and transactivates a series of antioxidant genes to scavenge the ROS as the cellular response to the oxidative stress [66, 67]. SPOP, another well-known substrate recognizing component of CRL3, was found to interact with INF2 (inverted formin 2) and promote its atypical ubiquitylation, leading to inhibition of INF2 ER localization instead of degradation. The dissociation of INF2 from ER reduced the formation of mitochondrial associated DRP1 puncta and abrogated mitochondrial fission. Biologically, INF2-mediated mitochondrial fission is involved in migration and invasion, the processes promoted upon mutational inactivation of SPOP in human prostate cancer cells [68].

### CRL4

CRL4 consists of cullin-4, RBX1, adaptor protein DDB1 (UV-damaged DNA-binding protein 1), and substrate receptors DCAFs (DDB1-CUL4-associated factor). Cullin-4 has two family members cullin-4A and cullin-4B, which share 82% sequence identity, but target different sets of substrates [69]. Cereblon (CRBN), a Cul-4A substrate receptor, was partially localized within the mitochondrial matrix. Instead of targeting degradation of Cul-4A substrates, CRBN acted as a Lon protease to suppress neuronal cell death upon induced by oxidative stress [70]. Moreover, CC-885, a novel thalidomide derivative that bridges the interaction between CRBN and its neosubstrate BNIP3L (also known as NIX), leading to ubiquitylation and degradation of BNIP3L to abrogate BNIP3L-dependent mitophagy [71]. Furthermore, a recent study showed that muscle-specific deletion of DCAF6, another substrate receptor of Cul-4A, resulted in reduced binding between Z-disc proteins ACTN2 and Cap-Z as well as increased levels of mitochondrial ROS and impaired respiration/ATP production in mouse hearts or cardiomyocytes isolated from these mice. The authors concluded that DCAF6 deficiency contributes to the pathogenesis of limb-girdle muscular dystrophy (LGMD) and heart failure, although detailed molecular mechanism remains elusive [72].

A direct evidence that CRL4B regulates mitochondrial function was shown by a Cul-4b knockout study. *Cul-4b* deletion in germ cells led to male infertility due to impaired sperm motility, partly caused by reduced mitochondrial activities including lower membrane potential and decreased ATP production. While the authors identified that Insl6, an insulin family member, is a novel substrate of Cul-4b in male germ cells, as possible mechanism, no functional rescue experiment was conducted to demonstrate that accumulation of Insl6 is indeed the cause of infertility derived from impaired mitochondrial function [73]. In another study, Arylhydrocarbon Receptor (AhR), a substrate receptor subunit in the CUL4B-AhR complex [69] was reported to protect melanocytes from oxidative damage via a mechanism that involved the up-regulation of nuclear respiratory factor 1 (NRF1) and its downstream targets to increase mitochondrial DNA synthesis and ATP production [74]. Furthermore, in human lung fibroblasts, genetic ablation of AhR decreased mitochondrial membrane potential and increased mitochondrial ROS to trigger cytochrome c release and subsequent apoptotic cell death when exposed to oxidative stress induced by tobacco extracts. Mechanistically, AhR appears to act as an antioxidant, rather than an E3 ligase for substrate degradation [75].

### CRL5

CRL5 consists of cullin-5, a RING finger protein RBX2, adaptor protein Elongin B/C, and SOCS-box containing receptor proteins [62]. Compared to many CRL5 substrates for targeted degradation [76, 77], Cullin-5 bound to TRAF6 and promoted its polyubiquitylation via K63 linkage in response to LPS exposure, which facilitated NF-κB activation to trigger inflammatory response [78]. Interestingly, on depolarized mitochondria, TRAF6 was found to form a complex with PINK1 and SARM1 to promote K63-linked ubiquitylation and stabilization of PINK1. As a result, parkin, a PINK1 partner, was recruited to damaged mitochondria to facilitate mitophagy [79]. DEPTOR, a naturally occurring inhibitor of both mTORC1 and mTORC2, has been identified as the substrate of both CRL1 (SCF^β−TrCP1^) and CRL5, respectively [80, 81]. One study showed that knockdown of DEPTOR in cultured term primary human trophoblast (PHT) cells promoted mitochondrial respiration, mtDNA copy number, and citrate synthase activity with mechanism involving activation of mTORC1 and mTORC2 [82].

The CRL7 and CRL9 were seldom studied family members of CRLs, and no reports were found in their involvement of mitochondrial regulation.

Taken together, the CRL regulation of mitochondria occurs by either promoting ubiquitylation and degradation of mitochondrial proteins or their regulators, or in a degradation independent manner via modulating other key molecules that affect mitochondrial functions. The finding that CRLs play an important role in modulating mitochondrial network indeed broadens our understanding of how mitochondria is precisely regulated.

### Non-cullin substrates

The neddylation substrates can be broadly classified into two categories: commonly studied cullins and less studied non-cullin substrates. Two studies reported potential regulation of non-cullin substrates in mitochondrial functions. A recent study showed that electron transfer flavoproteins (ETFs: ETFA and ETFB) were subjected to neddylation modification, which resulted in their stabilization by preventing ubiquitylation and degradation in hepatocytes and also partially facilitated fatty acid β-oxidation in neonatal mice. In the same vein, the mutants of ETFA and ETFB with neddylation site abrogated had reduced protein levels or activities which contributed to the pathogenesis of glutaric aciduria type II (GA-II). Furthermore, ETFA knockdown led to substantially decreased basal and maximal respiration [28].

The second study showed that HIF-1α and HIF-2α are subjected to neddylation modification, which led to their stabilization in a manner independent of prolyl hydroxylase (PHD)/VHL oxygen-sensing system, but dependent on mitochondria-generated ROS, although the biological consequence of such stabilization was not determined [83].

Furthermore, some non-cullin substrates were found to regulate mitochondrial function, but whether this is related to neddylation modification is unclear. For example, in response to various DNA damaging agents, p53, a non-cullin substrate of neddylation, was translocated to mitochondria to regulate apoptosis [84, 85]. Although p53 neddylation mediated by MDM2 or SCF^FBXO11^ inhibited its transcription activity [86, 87], whether its neddylation modification impacts mitochondrial functions remains unclear. One more case is Parkin, an E3 ubiquitin ligase, that promotes ubiquitylation and degradation of MFN1/2 to trigger mitophagy [88]. While Parkin is subjected to neddylation modification, which activated its ligase activity, but not altering its subcellular localization [89], there is no direct biological evidence to show that Parkin neddylation indeed enhanced mitophagy.

## Neddylation inhibitor MLN4924 alters mitochondrial morphology and function

Cellular ROS is mainly generated in mitochondrial powerhouse. Abnormal ROS overproduction due to impaired mitochondria alone or in combination with defective antioxidative scavenger systems contributes significantly to the damages to cellular DNA, proteins, and lipids, leading to tumorigenesis [90]. MLN4924 (also known as pevonedistat in the clinical trials), a small molecular inhibitor of catalytic subunit of NEDD8 activating enzyme, blocks the entire neddylation modification and consequently inactivates all CRLs [19]. Given overactivation of neddylation system and CRLs in a variety of human cancers, MLN4924 has been shown impressive anti-cancer activity in many preclinical studies and had advanced to few Phase II clinical trials as an anticancer agent alone or in combination with various chemotherapeutic drugs [22].

Several studies including from our own laboratory showed that MLN4924 regulates mitochondrial ROS production and other mitochondrial functions. In acute myeloid leukemia cells, MLN4924 inactivated CRL1/SCF to inhibit NF-κB via accumulated IκB, leading to increased ROS generation due to downregulation of Mn-SOD (superoxide dismutase), a typical NF-κB downstream gene and a major antioxidant enzyme to scavenge ROS. Thus, MLN4924-induced disruption of cellular redox status was identified as a key event in apoptosis induction [19, 30]. Moreover, other two studies reported that MLN4924 induced ROS generation by impairing mitochondrial membrane potential in both ovarian and live cancer cell lines to cause apoptosis through the upregulation of pro-apoptotic proteins, including PUMA, BIK, NOXA and BIM. Among these proteins, NOXA and BIK were identified as important downstream effectors for apoptosis induced by MLN4924/CQ and MLN4924/cisplatin combination, respectively. Importantly, the ROS scavenger reduced the expression of these pro-apoptotic proteins and attenuated apoptosis, indicating the causal effect of ROS [31, 91].

**Fig. 3:**
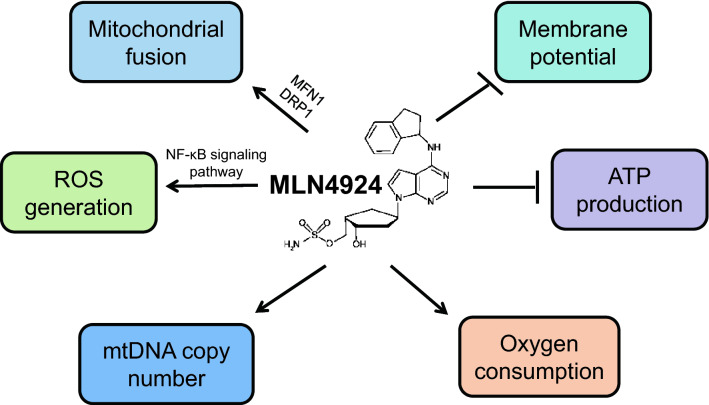
MLN4924 regulates mitochondrial functions. MLN4924 induces mitochondrial fission-to-fusion conversion, increases mitochondrial copy number, oxygen consumption and ROS production, but inhibits mitochondrial membrane potential and ATP production. The proteins and signaling pathways involved in MLN4924-regulated mitochondrial functions are shown [30, 35]

Recently, we found that MLN4924 induced mitochondrial fission-to-fusion conversion in time-and dose-dependent manners in a variety of human cancer cell lines. Mechanistic studies revealed that MLN4924 inactivated SCF^β−TrCP^ E3 ligase to cause accumulation of MFN1 [35], which is a protein previously known to trigger the fission-to-fusion conversion [92]. MLN4924 also reduced the level of phospho-DRP1^S616^ in mitochondria to increase cytoplasmic DRP1 content. The mitochondrial functional assays showed that MLN4924 inhibited TCA cycle but promoted both the basal and maximal oxygen consumption rate (OCR), while reducing the intracellular ATP production. MLN4924 also caused mitochondrial depolarization and increased mitochondrial ROS levels as well as mtDNA copy number. Since MLN4924-induced mitochondrial fusion was coupled with increased oxidative phosphorylation (OXPHOS), we then tested translational implication of this finding by combined treatment of MLN4924 with metformin, an inhibitor of mitochondrial complex I, to determine possible enhanced anticancer efficiency in breast cancer cells. The results indeed showed a synergistic effect both in vitro cell culture and in vivo xenograft models [35]. Thus, our strategy of inhibiting both neddylation modification and OXPHOS provides new avenues for targeted therapy in some types of tumor such as breast cancer. It is interesting to mention that MLN4924 effect on OXPHOS appears to be cell-type dependent. Two other studies showed that MLN4924 decreased both basal and maximal OCR in liver cancer cell line and mouse hepatocytes [28, 93]. Taken together, MLN4924 induces mitochondrial fission-to-fusion conversion, mitochondrial copy number, oxygen consumption and ROS production, but inhibits mitochondrial membrane potential and ATP production (Fig. 3). MLN4924 may, therefore, have unique application against human cancers with dysfunctional mitochondria.

## Conclusion and future perspectives

**Fig. 4:**
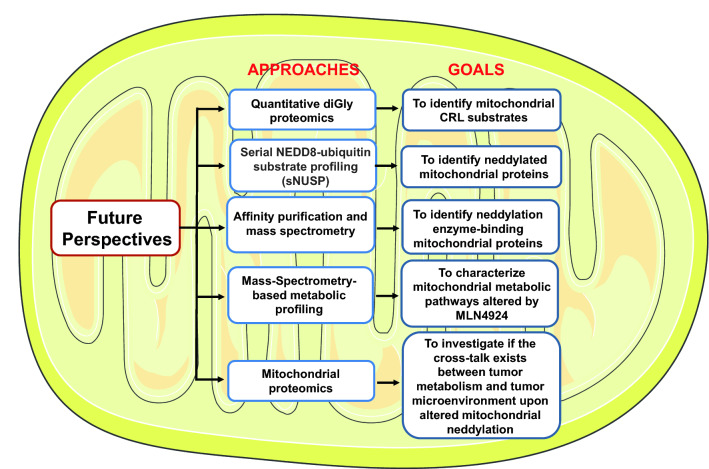
Proposed future directions for neddylation regulation of mitochondria. Five possible directions were proposed. See text for details

In this review, we summarized neddylation regulation of mitochondrial morphology and functions with potential anticancer application. The fact that several components and substrates of neddylation/CRLs, including NEDD8, UBA3, RBX1, FBXL4 and β-TrCP1, were found in mitochondria [28, 35, 54] provides the physical basis on neddylation regulation of mitochondria. The regulation is likely achieved via altered protein activity after neddylation modification or possible targeted degradation of mitochondrial substrates of CRLs. Nevertheless, neddylation regulation of mitochondria is still an emerging field in the broad area of mitochondria research with many unanswered questions. We propose the following aspects as future perspectives for this exciting field (Fig. 4).

### Identification of mitochondrial CRL substrates?

Mitochondrial proteins are subjected to degradation by both UPS and proteases. It is generally accepted that the UPS is involved in degradation of the outer membrane proteins, whereas the proteases are responsible for the cleavage and degradation of the proteins of inner mitochondrial compartments [94], although the UPS was also reported to be involved in the degradation of some cysteine-rich proteins in the intermembrane space (e.g. yeast Cox12), the uncoupling protein in the inner membrane, and a subunit of succinate dehydrogenase in the matrix [10, 94]. Several components of neddylation and CRLs were found in the mitochondria [28, 35, 54]. An open question is whether they actually assembly the active CRLs to promote the ubiquitylation of mitochondrial proteins for degradation via the UPS which is mainly localized outside the mitochondria? Given that few mitochondrial proteins were reported as the substrates of CRL, such as MFN1 by SCF^β−TrCP^ [35] and HAX-1 by SCF^FBXO25^ [61], and a comprehensive quantitative diGly proteomics analysis identified ~ 150 mitochondria proteins [95], it is likely that some mitochondrial proteins are indeed the substrates of CRLs. Here we propose that a similar analysis could be performed using HEK293 cells with knocked-in ubiquitin tagged with a mitochondrial signal peptide at the N-terminus, followed by mitochondrial purification and diGly proteomics. The approach will validate previous candidates with identification of additional putative mitochondrial substrates of CRLs for further confirmation and functional characterization.

### Identification of neddylated mitochondrial proteins

Only one study reporting that mitochondrial protein parkin is subjected to neddylation modification which increased its E3 ligase activity [89]. To identify almost, if not all, mitochondrial proteins subjected to neddylation modification at the whole mitochondria level, an approach similar to serial NEDD8-ubiquitin substrate profiling (sNUSP), reported recently [20] can be employed. Specifically, the approach includes the establishment of a knock-in HEK293 cell line of NEDD8 R74K mutant tagged with a mitochondrial signal peptide at the N-terminus for mitochondria targeting, followed by mitochondrial isolation, Lys-C digestion, K-εGG-peptide enrichment, and finally Mass Spectrometry analysis to identify neddylated mitochondrial proteins. The specificity can be determined by including a MLN4924-treatment control group. Each validated candidate can be further characterized for functional significance of neddylation modification.

### Identification of mitochondrial proteins that bind to neddylation enzymes

To mechanistically elucidate neddylation regulation of mitochondrial function, it should be meaningful to isolate mitochondrial proteins that bind to any of neddylation enzymes. Again, a given neddylation enzyme expression construct can be made with the N-terminal tag of mitochondrial signal peptide and the C-terminal FLAG-tag for affinity purification. The construct can then be knocked-in to HEK293 cells, and selected stable clone to be used for routine affinity purification and mass spectrometry, followed by validation of the candidates in an individual basis and biological effect on mitochondrial function determined.

### Characterization of mitochondrial metabolic pathways altered by MLN4924

We recently performed Mass-Spectrometry-based metabolic profiling of breast cancer cells to investigate the overall effect of MLN4924 on cell metabolism at a global level, and found that MLN4924 treatment caused significant increase or decrease of many metabolites in cell extracts [35]. Similarly, integrated liquid chromatography-tandem mass spectrometry (LC–MS/MS) analysis of metabolites and mass spectrometry-based proteomic analysis of proteins should be performed using isolated mitochondria upon overall neddylation inhibition (by MLN4924) or manipulation of each individual cullins to define specifically the effect of neddylation/CRLs on mitochondrial metabolism.

### The cross-talk between tumor metabolism and tumor microenvironment: involvement of mitochondrial neddylation?

Increasing amount of data has shown that neddylation can modulate both tumor metabolism and tumor microenvironment [18, 35]. Whether and how mitochondria are involved in this cross-talk upon regulation by neddylation/CRLs is an interesting topic for future investigation. As an initial step, mitochondrial proteomics could be performed under the conditions in which tumor metabolism and tumor microenvironment are altered upon MLN4924 treatment to determine potential involvement of mitochondrial neddylation.

## Data Availability

Not applicable.
